# Affordability of Adult Tuberculosis Vaccination in India and China: A Dynamic Transmission Model-Based Analysis

**DOI:** 10.3390/vaccines9030245

**Published:** 2021-03-11

**Authors:** Chathika Krishan Weerasuriya, Rebecca Claire Harris, Matthew Quaife, Christopher Finn McQuaid, Richard G. White, Gabriela B. Gomez

**Affiliations:** 1 TB Modelling Group, TB Centre and Centre for the Mathematical Modelling of Infectious Diseases, Department of Infectious Disease Epidemiology, Faculty of Epidemiology & Population Health, London School of Hygiene, London WC1E 7HT, UK; rebecca.harris@lshtm.ac.uk (R.C.H.); matthew.quaife@lshtm.ac.uk (M.Q.); finn.mcquaid@lshtm.ac.uk (C.F.M.); richard.white@lshtm.ac.uk (R.G.W.); 2 COVID-19 Medical Franchise, Sanofi Pasteur, Singapore 189767, Singapore; 3 Department of Global Health & Development, Faculty of Public Health & Policy, London School of Hygiene and Tropical Medicine, London, WC1E 7HT, UK; gabriela.gomez@lshtm.ac.uk; 4 Department of Modelling, Epidemiology and Data Sciences, Sanofi Pasteur, 69007 Lyon, France

**Keywords:** tuberculosis, vaccine, model, affordability, budget, cost-effectiveness

## Abstract

New tuberculosis vaccines have made substantial progress in the development pipeline. Previous modelling suggests that adolescent/adult mass vaccination may cost-effectively contribute towards achieving global tuberculosis control goals. These analyses have not considered the budgetary feasibility of vaccine programmes. We estimate the maximum total cost that the public health sectors in India and China should expect to pay to introduce a M72/AS01_E_-like vaccine deemed cost-effective at country-specific willingness to pay thresholds for cost-effectiveness. To estimate the total disability adjusted life years (DALYs) averted by the vaccination programme, we simulated a 50% efficacy vaccine providing 10-years of protection in post-infection populations between 2027 and 2050 in India and China using a dynamic transmission model of *M. tuberculosis*. We investigated two mass vaccination strategies, both delivered every 10-years achieving 70% coverage: Vaccinating adults and adolescents (age ≥10y), or only the most efficient 10-year age subgroup (defined as greatest DALYs averted per vaccine given). We used country-specific thresholds for cost-effectiveness to estimate the maximum total cost (C_max_) a government should be willing to pay for each vaccination strategy. Adult/adolescent vaccination resulted in a C_max_ of $21 billion (uncertainty interval [UI]: 16–27) in India, and $15B (UI:12–29) in China at willingness to pay thresholds of $264/DALY averted and $3650/DALY averted, respectively. Vaccinating the highest efficiency age group (India: 50–59y; China: 60–69y) resulted in a C_max_ of $5B (UI:4–6) in India and $6B (UI:4–7) in China. Mass vaccination against tuberculosis of all adults and adolescents, deemed cost-effective, will likely impose a substantial budgetary burden. Targeted tuberculosis vaccination, deemed cost-effective, may represent a more affordable approach.

## 1. Introduction

New tuberculosis vaccines have made substantial progress in the clinical development pipeline [1]. The phase IIb trial of M72/AS01_E_ reported vaccine efficacy of 49.7% at 36 months in preventing pulmonary tuberculosis in adults with a previous history of tuberculosis infection [2]. Previous modelling studies suggest that new tuberculosis vaccines, particularly when delivered to adults or the elderly, may substantially contribute towards achieving global tuberculosis control targets [3,4,5].

These findings have renewed interest in deploying anti-tuberculosis vaccination to support achieving global tuberculosis control goals [6]. Previous studies of new or repurposed tuberculosis vaccines have consistently projected that vaccination is likely to be cost-effective [3,7].

Most studies that analysed cost-effectiveness have used willingness-to-pay (WTP) thresholds that only indicate if an intervention is good (or poor) value for money at the margin [8]. Historically, such analyses have used demand-side thresholds (e.g., multiples of gross domestic product per capita). Demand-side thresholds have been criticized for not reflecting country-level affordability of interventions. Alternative methods have been recently proposed, such as supply-side country-level WTP thresholds based on the marginal productivity of their respective healthcare systems [9,10].

Despite these developments, most studies that establish cost-effectiveness have not assessed the affordability of vaccination programmes [11]. Affordability is particularly relevant to vaccination programmes, which while often cost-effective, must be deployed at a large scale. Discrepancies between cost-effectiveness and affordability may arise due to the methods of WTP threshold estimation. Demand-side thresholds are typically exogenously determined, without consideration of other interventions provided by the health system, the overall healthcare budget, or the different timing of costs and benefits of vaccination. Supply-side WTP thresholds, although linked to local healthcare budgets, have raised concerns of transferability across settings. Widely cited examples of such conflicting results include the provision of directly acting antiviral drugs for Hepatitis C [12,13] and the use of Gene Xpert rapid molecular diagnostic testing for tuberculosis [14,15].

Tuberculosis vaccination delivered to adults is likely to require large-scale mass campaigns. This represents a very large target population for two of the countries with the greatest number of incident TB cases: China and India. While previous studies have found that adult and elderly TB vaccination may be cost-effective [3,7], no studies have investigated whether mass vaccination programmes costed based on their maximum cost-effective value to the healthcare systems could be affordable.

In this study, we estimate the maximum cost of a cost-effective, large-scale adult TB vaccine programme in China and India, assuming country-specific willingness to pay per DALY averted thresholds under varying vaccine implementations. Finally, we discuss the affordability implications of these estimates in the context of tuberculosis-specific and general expenditure by the public health sectors of India and China.

## 2. Materials and Methods

### 2.1. Transmission Model

We developed an age-, treatment-history and drug-resistance stratified compartmental dynamic transmission model of tuberculosis. The model, parameters, calibration and data sources are described in full elsewhere [7]. Briefly, uninfected (susceptible) individuals who acquired *Mycobacterium tuberculosis* (M. tb) infection could transition directly to active (infectious or non-infectious disease) or to latent tuberculosis infection (LTBI). Individuals with LTBI could reactivate to active disease. From active disease, individuals could be initiated on TB treatment, self-cure or die. Following successful treatment or self-cure, individuals transitioned to the resolved state from where they could relapse back to active disease. Individuals could also be infected by drug-susceptible TB and develop drug-resistance on treatment or be infected by drug-resistant M. tb. A diagram detailing model compartments and flows is presented in Weerasuriya et al. [7] (additional File 1, Figure S1). We calibrated the model to historical epidemiologic data in India and China from 2000 to 2018 and projected TB epidemiology over 2018 to 2050, in line with TB control milestones and timelines specified in the WHO End TB goals [16,17,18]. In the baseline (no-vaccine) scenario, programmatic management of TB was unchanged after 2018. Final projections were derived through the aggregation of results from 1000 fully calibrated parameter sets.

### 2.2. Vaccine Implementation

We simulated vaccination from 2027 onwards and compared the vaccine-enabled model output with corresponding unvaccinated baseline model output to estimate vaccine impact over 2027–2050. We assumed characteristics aligned with M72/AS01_E_ and modelled a vaccine with 50% efficacy conferring 10-years of protection. The vaccine protected against disease, but not infection by M. tb, and was assumed to be effective only in those with a previous history of infection by M. tb (referred to as a “post-infection” efficacy vaccine). We did not explicitly model existing neonatal Bacillus Calmette–Guérin (BCG) immunisation programmes, as the effect of BCG was assumed to be reflected in the baseline epidemiology through model calibration targets. The vaccine was administered to individuals with neither active disease nor who were receiving treatment for TB and mediated its effect through reducing the rate of progression to active disease following infection and reducing the rate of reactivation or relapse from latent disease or a recovered state. Vaccine waning was assumed to be instant at the end of the duration of protection.

We investigated two scenarios of adult and adolescent mass vaccination: “all age” and “targeted”.

#### 2.2.1. All-Age Vaccination

In all age vaccination, vaccination was delivered to all ages ≥10 years through mass campaigns delivered in 2027, 2037 and 2047. Vaccines were assumed to be delivered within a single calendar year. As there was no direct analogue for all-age mass vaccination of adults in India or China on which to base probable vaccine coverage, we assumed a value of 70% based on a composite of sources: (a) Menafrivac campaigns delivered to 1–29-year-olds in South Africa, which achieved coverage of 70–98% [19]; (b) routine influenza vaccination in China [20,21], achieving coverage of 36–49%; and (c) Japanese encephalitis mass adult vaccination campaigns in India [22], achieving coverage of 58%. Both influenza and Japanese encephalitis campaigns were delivered to populations that included the elderly. Since the all-age vaccination strategy we modelled also included the elderly, we opted for the lower estimate of Menafrivac coverage but performed sensitivity analyses by simulating coverage of 10% and 90%.

#### 2.2.2. Targeted Vaccination

In the “targeted” scenario, vaccination was delivered to a 10-year wide age band through similar mass campaigns. For each country, this group was chosen by segmenting the population between 10 and 99 years into 10-year wide groups and identifying the group with the highest vaccination efficiency (defined as the number of DALYs averted per vaccine given). We assumed vaccine coverage of 70% to maintain comparability to the “all-age” strategy.

### 2.3. Vaccine Programme Cost Estimation

#### 2.3.1. TB Programme Cost Model

We used an ingredients-based approach to estimate the (non-vaccine) TB control programme costs from the public healthcare sector perspective. We applied country-specific unit costs to diagnosis, including drug-sensitivity testing and treatment for both drug-sensitive and drug-resistant TB. For India, we additionally included costs borne by the public healthcare sector for nutritional support to TB patients on treatment and incentive payments to the private healthcare sector. A full description of unit costs, including sources, is given in Weerasuriya et al. [7]. The cost-model was restricted to direct costs to the TB programme.

#### 2.3.2. Costs and Benefits of Vaccines and Vaccine Programmes

We measured the health benefit of vaccination in disability-adjusted life years (DALYs) lost by tuberculosis. DALYs were calculated by applying disability weights for tuberculosis per the Global Burden of Disease study [23] and conditional life expectancy per the UN World Population Prospects [24]. The effectiveness of vaccination was then estimated as DALYs averted comparing the vaccine scenario to the baseline (non-vaccine) scenario. 

We estimated the maximum total cost of a vaccination programme the public health sector should be willing to pay, thus the intervention remained cost-effective given our assumed vaccine characteristics and implementation strategies. The maximum total cost of a vaccination programme (C_max_) was calculated as follows:

(1) $$C_{\max}=\left(\mathrm{WTP}\times \Delta \mathrm{DALYs}\right)-\left(C_{V}-C_{B}\right)$$

Where WTP is the willingness to pay threshold per DALY averted; ΔDALYs are the DALYs averted comparing the vaccine scenario to the baseline (non-vaccine) scenario; and C_v_ and C_B_ are the total costs of the TB programme in the vaccine and baseline scenarios, respectively. The term WTP×ΔDALY represents the monetary value of DALYs averted by vaccination, whereas the term (C_V_-C_B_) represents the net cost-savings in the TB programme due to reduced TB burden. C_max_ represents the maximum incremental cost the buyer should be willing to pay for vaccination. The incremental cost-effectiveness ratio for vaccination is equal to the willingness-to-pay threshold. A particular vaccine implementation that averted more DALYs would lead to a higher C_max_ value, reflecting greater value provided to the health system.

We also calculated the maximum total cost per course of vaccination (including dose, delivery and programmatic costs) that the public health sector should be willing to pay, subject to assumptions above, as C_max_ divided by the number of vaccines delivered. For a given WTP threshold, a higher cost per vaccine course was interpreted as the additional cost per course that a payer should be willing to pay for the greater number of DALYs averted per vaccine course.

The country-specific willingness-to-pay thresholds were taken from Ochalek et al. [9]. This study estimated the elasticity of mortality outcomes, survival and morbidity burdens of disease with respect to public healthcare expenditure to derive the estimated number of DALYs averted by a 1% change in healthcare expenditure. The reciprocal of this value­—the cost per DALY averted—estimated the marginal productivity of the health system, i.e., the opportunity cost of healthcare spending on a given intervention. These values represented a supply-side estimate of willingness-to-pay per DALY averted grounded in country-specific healthcare expenditure and budgets. Ochalek el al. provided 4 estimates per country, reflecting slightly different assumptions in their calculations. In this analysis, we used the lowest and highest estimates, for India estimated at $264 and $363 and for China at $3650 and $5669 per DALY averted, respectively. Given that WHO now encourages the use of country-based thresholds instead of GDP-based thresholds [8], and given that the country-specific thresholds are substantially lower than GDP per capita estimates for both India and China, demand-side thresholds based on GDP per capita were excluded from our analysis.

We discounted costs and health benefits at 3% to 2018 values in the base case analysis, per standards from the Gates reference case for economic evaluation [25]. Rates were set at 3% in both India and China to ensure comparability. Undiscounted results were presented as a sensitivity analysis in supplementary material section 1.1, Figures S1 and S2, and Table S1. All costs are presented in USD.

### 2.4. Baseline Scenario Analysis

To capture uncertainty in future health system investments, we defined an alternative “Policy” baseline scenario, representing a scale-up of programmatic TB management for each country.

For China, the Policy scenario was informed by country expert opinion [7] and comprised 2 changes. First, we linearly scaled-up drug-susceptibility testing coverage to 90% from 2018 to 2036. Second, we introduced a standard 9-month RR/MDR-TB treatment besides the baseline 24-month regimen, maintaining the same treatment success rate across both regimens. The proportion of drug-resistant TB treated with the 9-month regimen was linearly increased to 40% of all second-line therapy from 2018 to 2036.

For India, we used the National Strategic Plan of the Indian Revised National Tuberculosis Control Programme [26] to inform the Policy scenario. We implemented three changes: (a) Increased case detection rate (combined across private and public sectors) from approximately 60% to 85%; (b) increased drug-susceptibility testing coverage among public sector notifications to 100%; and (c) increased proportion of notifications originating from the private sector to 35%, all from 2018 to 2025.

### 2.5. Patient and Public Involvement

Patients were not involved in the conduct of this study.

## 3. Results

### 3.1. All-Age Vaccination

#### 3.1.1. Averted Burden

A post-infection 50% efficacy vaccine conferring 10-years of protection, delivered to all adults (aged ≥10 years) at 10-yearly intervals at coverages of 10%, 70% and 90%, was predicted to avert 8.3 (Uncertainty Interval [UI]: 6.5–10.5) million, 52.7 (UI 42.8–65.2) million and 65.7 (UI 53.6–80.9) million DALYs by 2050 in India, corresponding to 0.021 (UI 0.016–0.026), 0.019 (UI 0.015–0.023) and 0.018 (UI 0.015–0.023) DALYs averted per vaccine delivered.

In China, vaccination at coverages of 10%, 70% and 90%, was predicted to avert 0.5 (UI 0.4–0.7) million, 3.8 (UI 3.0–4.9) million and 4.8 (UI 3.8–6.3) million DALYs by 2050, corresponding to 0.001 (UI 0.001–0.002), 0.001 (UI 0.001–0.002) and 0.001 (UI 0.001–0.002) DALYs averted per vaccine delivered.

#### 3.1.2. Maximum Vaccination Programme Cost

**Figure 1:**
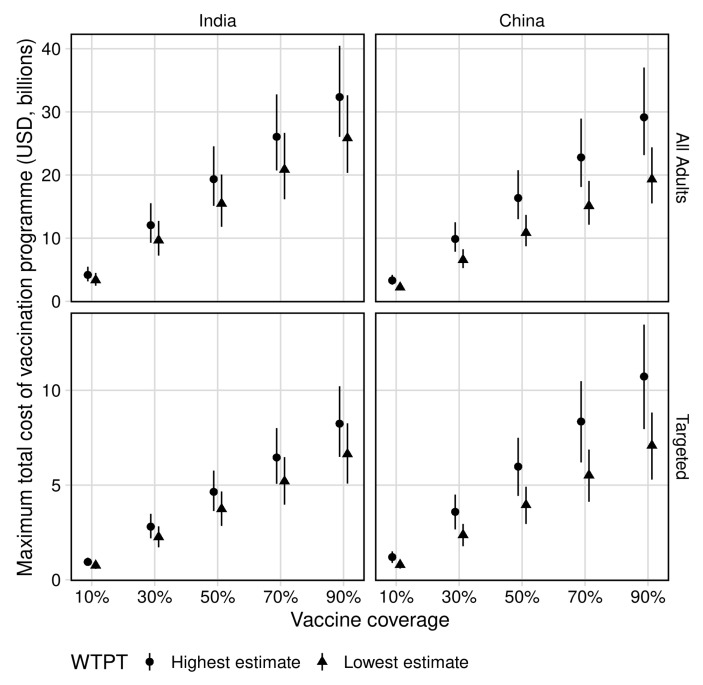
Maximum total vaccine programme cost. Top panels represent all-age vaccination (adults ≥10 years); bottom panels represent targeted vaccination (ages 50–59 in India and ages 60–69 in China). WTPT = willingness to pay thresholds per Ochalek et al. [9], estimated at $264 and $363 per DALY averted in India (lowest and highest estimates, respectively) and $3650 and $5669 per DALY averted in China (lowest and highest estimates, respectively). Costs discounted to 2018 values.

**Figure 2:**
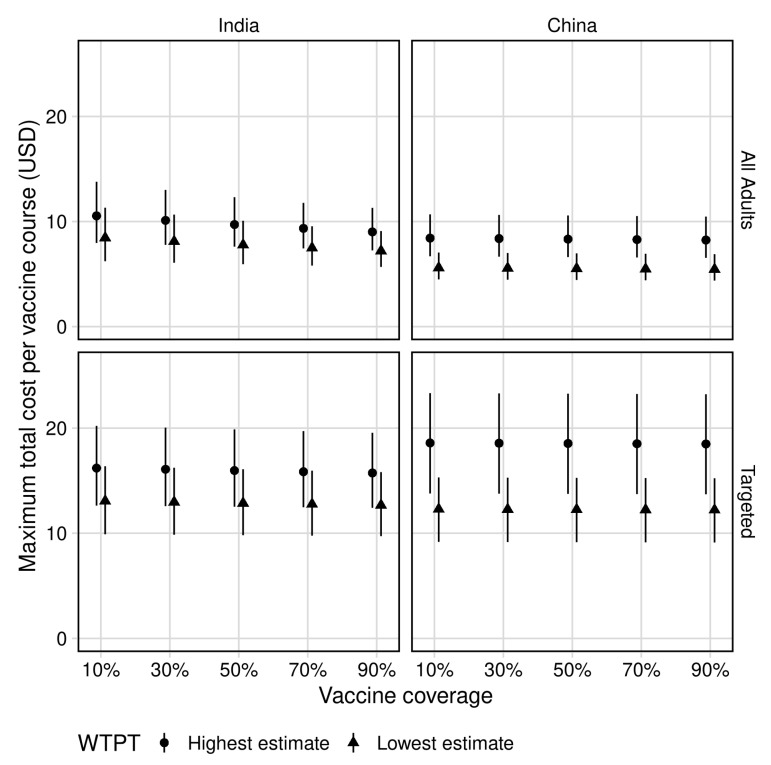
Maximum total cost per vaccine course. Top panels represent all-age vaccination (adults ≥10 years); bottom panels represent targeted vaccination (ages 50–59 in India and ages 60–69 in China). WTPT = country-specific willingness to pay thresholds per Ochalek et al. [9], estimated at $264 and $363 per DALY averted in India (lowest and highest estimates, respectively) and $3650 and $5669 per DALY averted in China (lowest and highest estimates, respectively). Costs discounted to 2018 values.

The maximum total cost of a vaccination programme for all age vaccination to remain cost-effective and the predicted total cost per vaccine course are presented in Table 1, Figure 1 and Figure 2, respectively.

In India, mass vaccination of all adults at 70% coverage was predicted to cost up to $21 (UI 16–27) billion and $26 (UI 21–33) billion at the lower and upper WTP thresholds of $264 and $363 per DALY averted, respectively. This corresponded to a maximum total cost per course of $7 (UI 6–10) and $9 (UI 7–12) for the approximately 3 (UI 3–3) billion vaccines delivered to remain cost-effective.

In China, mass vaccination of all adults at 70% coverage was predicted to cost up to $15 (UI 12–19) billion and $23 (UI 18–29) billion at the lower and upper WTP thresholds of $3650 and $5669 per DALY averted, respectively. This corresponded to a maximum total cost per course of $5 (UI 4–7) and $8 (UI 7–11) for the approximately 3 (UI 3–3) billion vaccines delivered to remain cost-effective.

### 3.2. Targeted Vaccination

#### 3.2.1. Optimal Target Age Groups and Averted Burden

**Figure 3:**
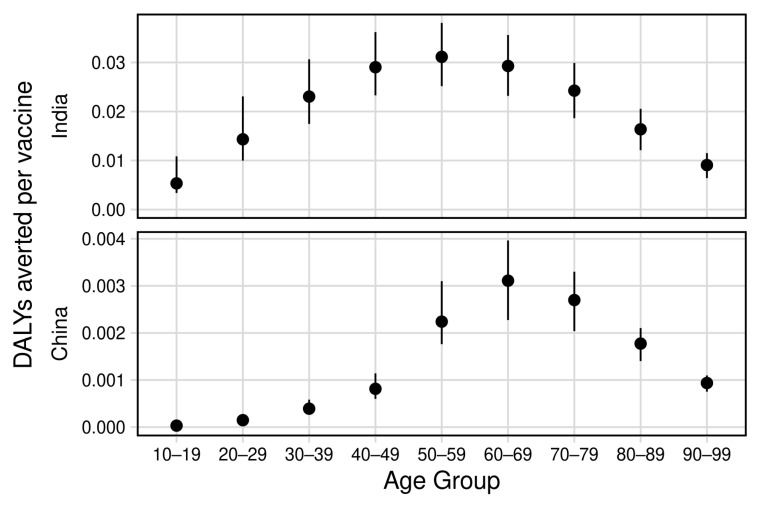
Vaccination efficiency by target age group. Efficiency is defined as the number of DALYs averted per vaccine delivered. Mass vaccine campaigns were deployed at 70% coverage to each age group.

The predicted efficiency of vaccination (in terms of DALYs averted per vaccine delivered) is presented in Table 1 and Figure 3. Age-specific TB prevalence in the underlying transmission model is presented in supplementary material section 1.2, Figures S3 and S4.

In India, we found that vaccination of 50–59-year-olds was most efficient (Figure 3), with 70% vaccine coverage averting 12.7 (UI 10.3–15.5) million DALYs by 2050, corresponding to 0.031 (UI 0.025–0.038) DALYs averted per vaccine delivered over 407 (UI 405–410) million vaccines. Vaccination of the adjacent age groups 40–49 and 60–69 years yielded similar efficiencies of 0.031 (UI 0.025–0.038) and 0.031 (UI 0.025–0.038) DALYs averted per vaccine delivered, respectively.

In China, we found that the most efficient age group for vaccination was older, between 60–69-year-olds, averting 1.4 (UI 1.0–1.8) million DALYs by 2050, corresponding to 0.003 (UI 0.002–0.004) DALYs averted per vaccine delivered over 451 (UI 451–452) million vaccines. In contrast to India, the optimum age group was more sharply defined, with a greater difference compared to vaccination of adjacent age groups (Figure 3). Fewer DALYs were averted per vaccine delivered across all age-groups in China compared to India.

#### 3.2.2. Maximum Vaccination Programme Cost

The maximum total cost of a vaccine programme for targeted vaccination to remain cost-effective and the predicted total cost per vaccine course are presented in Table 1, Figure 1 and Figure 2, respectively.

In India, mass vaccination of adults aged 50–59 at 70% coverage was predicted to cost up to $5 (UI 4–6) billion and $6 (UI 5–8) billion at the lower and upper WTP thresholds of $264 and $363 per DALY averted, respectively. This corresponded to a maximum total course per vaccine course of $13 (UI 10–16) and $16 (UI 12–20) for the approximately 407 (UI 405–410) million vaccines delivered to remain cost-effective.

In China, mass vaccination of adults aged 60–69 at 70% coverage was predicted to cost up to $6 (UI 4–7) billion and $8 (UI 6–10) billion at the lower and upper WTP thresholds of $3650 and $5669 per DALY averted, respectively. This corresponded to a total cost per vaccine course of $12 (UI 9–15) and $19 (UI 14–23) for approximately 451 (UI 451–452) million vaccines delivered to remain cost-effective.

### 3.3. Policy Scenario Analysis

In India, when (non-vaccine) TB programme activities were scaled up, targeted vaccination at 70% coverage was predicted to cost up to $5 (UI 4–7) billion and $4 (UI 3–5) billion at the upper and lower WTP thresholds, respectively, corresponding to a maximum total cost per vaccine course of $13 (UI 10–16) and $11 (UI 8–13). In China, targeted vaccination at 70% coverage was predicted cost up to $8 (UI 6–10) billion and $5 (UI 4–7) billion at the lower and upper WTP thresholds, respectively, corresponding to a maximum total cost per vaccine course of $18 (UI 13–23) and $12 (UI 9–15). In both countries, the maximum costs for a cost-effective vaccination programme in the policy scenario were lower than for the baseline scenario without TB programme scale-up, reflecting a lower avertible burden of TB by vaccination. This effect was substantially smaller in China than India. This reflected the future TB programme scale-up strategy, which in China focused on increased case detection and treatment of drug-resistant tuberculosis, while in India was focussed on both drug-sensitive and drug-resistant tuberculosis. Complete results for the policy scenario are given in supplementary material section 1.3, Figures S5–S9, and Tables S2 and S3.

## 4. Discussion

Cost-effective mass vaccination of adults and adolescents aged ≥10 years against tuberculosis in India was estimated to cost $26B and $21B at high ($363/DALY averted) and low ($264/DALY averted) willingness-to-pay (WTP) thresholds, respectively, over 2027–2050, corresponding to a total cost per vaccine course (including dose, delivery and programmatic costs) of $9 and $7, respectively. In China, cost-effective mass vaccination using the same strategy was predicted to cost $23B and $15B at high ($3650/DALY averted) and low ($5669/DALY averted) WTP thresholds, respectively, corresponding to a total cost per vaccine course of $8 and $5, respectively. Cost-effective mass vaccination of a targeted high-efficiency age group was predicted to incur lower total costs but higher costs per course of vaccine than mass vaccination of all ages. In India, vaccinating ages 50–59 was predicted to cost $6B and $5B at the high and low thresholds, respectively, over 2027–2050, corresponding to a total cost per course of $16 and $13. In China, vaccinating ages 60–69 was predicted to cost $8B and $6B at the high and low thresholds, respectively, by 2050, corresponding to a maximum total cost per vaccine course of $19 and $12.

Increased investment in the (non-vaccine) TB programme led to substantial reductions in the estimated maximum vaccination programme costs in India, with moderate reductions in China. This follows from lower TB case detection rates in India [16] than China of both drug-sensitive and drug-resistant TB at baseline, which translates into a greater burden of averted disease due to programmatic scale up. Moreover, the planned scale-up of programmatic TB management was greater in India than in China.

Each vaccine course averted more DALYs and delivered more value to the health system in the targeted vaccination scenario compared with the “all-age” scenario. Therefore, at a given willingness to pay threshold, the total cost per vaccine course that the health system should be willing to pay was correspondingly higher in the targeted than all age scenarios. Assuming the vaccine price borne by the health system is independent of the implementation scenario, this would translate to proportionately more funds available for programmatic aspects of vaccination (e.g., logistics, campaign organisation).

### 4.1. In Context

Our study suggests that an all-age adult and adolescent mass tuberculosis vaccination programme, considered cost-effective at country-specific cost-effectiveness thresholds, would impose a substantial budgetary burden on the health system in India and China. We estimated the maximum cost of such a vaccination programme at the lowest WTP threshold to be $21B (UI 16–27) and $15B (UI 12–19) in India and China, respectively, for three vaccination campaigns in 2027, 2037 and 2047. In comparison, annual universal infant vaccination programmes in India and China are estimated to cost approximately $700 million (2013–14 levels, adjusted to 2017 prices) [27] and $1B (2015 levels) [28], respectively, while the World Health Organization estimates the total annual budgets available for national strategic plans for tuberculosis in India and China at $583 million and $719 million in 2019, respectively. In this context, targeted vaccination may represent a more affordable approach. The maximum cost of a cost-effective vaccination programme targeted to the highest efficiency 10-year age group was estimated at between $5B (UI 4–6) and $6B (UI 4–7) in India and China, respectively, for three vaccination campaigns over 2027–2050. While routine infant vaccination is not a direct comparator to spaced adult and adolescent mass vaccination campaigns, this approximate comparison suggests that the maximum estimated costs of cost-effective targeted TB vaccination may be comparable to existing and funded health interventions. However, routine infant vaccination provides multiple doses against multiple conditions per fully immunised child.

Our findings suggest that cost-effectiveness estimates, even with supply-side WTP thresholds, will likely provide an incomplete assessment of the economic feasibility of mass TB vaccination in India and China to decision makers, as they do not reflect the probable budget impact of such a programme. Further, marginal WTP thresholds may inadequately capture the health opportunity costs of interventions, which incur very high expenditures [29]. Decision makers should, therefore, carefully assess the appropriateness of WTP thresholds used in the economic evaluation of TB vaccines. This study also demonstrates the use of a dynamic model to identify a high-efficiency subgroup for vaccine targeting. While age alone may be an insufficient criterion on which to base targeting strategies, this approach may be generalisable to identify other risk groups or stratifications, which can maximise efficiency.

We present total cost-estimates of national vaccination programmes in both India and China. Both countries have complex health systems with healthcare provisions devolved to subnational administrative divisions, with variation in TB epidemiology, costs of providing programmatic TB management and immunisation services. Regional affordability of mass vaccination is likely to be sensitive to these local factors. Future studies could explore such factors to assess affordability at the regional level.

### 4.2. Willingness to Pay Thresholds

In this study, we used healthcare opportunity cost-based willingness to pay thresholds substantially lower than demand-side WTP thresholds (for example, based on GDP per capita) for India and China. If country decision makers were to adopt thresholds higher than the supply-side estimates we used in decision-making around tuberculosis vaccine programme financing, our cost estimates would be conservative. However, given that our total vaccination programme costs estimates were still substantial, this suggests that TB vaccines or vaccination cost-effective at GDP thresholds would be infeasible without substantial increases in healthcare expenditure beyond current levels, particularly in India.

### 4.3. Strengths and Limitations of This Study

We highlight two main limitations in our estimates of vaccine impact: (a) Paucity of data to substantiate vaccine deployment scenarios and their costs; (b) limitations in the deployed vaccine scenario.

There is no existing precedent for large, national, all-adult and adolescent vaccination campaigns in India or China. Assumptions around achievable coverage (particularly by age) are extrapolated from experience in other settings or age groups. We implemented mass vaccine campaigns within a single year rather than as a gradual scale-up of coverage and presented the maximum total cost of vaccination over three mass campaigns. The budget impact of the vaccination programme during each one of these three campaign years is considerable and not directly comparable to savings in the non-vaccine TB programme during inter-campaign years. Despite recent examples of COVID-19 vaccination rates, large-scale adult mass vaccination for tuberculosis may be delivered in multi-year campaigns. This would distribute budget impact over time. Vaccine waning is modelled as instant at the end of the duration of protection. This underestimated ongoing waning during inter-campaign years and during phased multi-year campaigns, leading to a likely overestimate of averted burden and of total maximum cost.

Our estimates of vaccine efficiency by 10-year age groups may also be affected by the lack of age-specific TB burden data. The underlying transmission model [7] was calibrated to age-specific data (children aged <15 years and adults aged ≥15 years) in both India and China. We found good concordance between empirical and modelled TB prevalence estimates by 10-year age group in China (Figure S1). However, nationally representative age-specific empirical estimates for India do not currently exist. Although TB prevalence predicted by the model for India (Figure S2) is consistent with *a priori* assumptions regarding the age-distribution of disease burden, these may be an over- or under-estimate. Without further data, it is difficult to deduce how this might bias our estimated total cost of “targeted” mass vaccination.

We selected an age-group for the “targeted” implementation scenario based solely on vaccine efficiency. Total cost estimates may be an underestimate, as our analysis does not consider other priorities, e.g., targeting at-risk groups or equity considerations, which are often important features of vaccination programmes. As there is no data to inform this relationship, we assume that vaccine efficacy is invariant with age. Higher rates of vaccine failure and poorer vaccine responses are reported for influenza, pneumococcal and herpes zoster vaccines, amongst others [30]. If such a phenomenon were reported for new TB vaccines, the highest efficiency age groups may change.

A strength of this study is that we make no assumptions regarding the composition of vaccination programme costs (e.g., unit or programmatic costs), nor how this composition might vary with implementation (e.g., at different coverage levels, or by targeting to specific age groups). By modelling vaccines through a dynamic transmission model, we capture both the direct and indirect (transmission) effects of vaccination on TB burden and the corresponding impact on changes to the non-vaccine TB programme, which are incorporated into our estimates of total cost. Furthermore, our estimates incorporated the (averted) costs of drug-resistant tuberculosis: The underlying transmission model included a fully dynamic representation of resistance acquisition and transmission. The model was calibrated to rifampicin-resistant and multidrug-resistant tuberculosis (RR/MDR-TB) incidence and notification data in India and China. The underlying cost-model included country-specific estimates of RR/MDR-TB diagnosis and treatment costs and drug-sensitivity testing costs.

## 5. Conclusions

We found that cost-effective mass vaccination against tuberculosis of all adults and adolescents will likely impose a substantial budgetary burden on health systems in India and China. Cost-effective targeted tuberculosis vaccination, for example, by age, may represent a more affordable approach while also allowing greater expenditure per vaccine course delivered.

## Figures and Tables

**Table 1:** Mass vaccine campaigns. Targeted vaccination in India was delivered to ages 50–59 and in China to ages 60–69. Results are aggregated over three campaigns delivered in 2027, 2037 and 2047. Averted DALYs and estimated net vaccine implementation costs are discounted to 2018 values at 3% per year. WTP: Willingness to pay.

Country	Campaign	Averted DALYs (Total) ^a^	Averted DALYs (per Vaccine)	Vaccinations Delivered ^b^	WTP Threshold (DALY Averted)	Maximum Total Vaccine Programme Cost	Maximum Total Cost per Vaccine Course
India	All Ages	52.67M (42.79–65.18)	0.019 (0.015–0.023)	2.79B (2.78–2.80)	$264	$21B (16–27)	$7 (6–10)
					$363	$26B (21–33)	$9 (7–12)
	Targeted	12.69M (10.27–15.46)	0.031 (0.025–0.038)	0.41B (0.41–0.41)	$264	$5B (4–6)	$13 (10–16)
					$363	$6B (5–8)	$16 (12–20)
China	All Ages	3.79M (2.96–4.89)	0.001 (0.001–0.002)	2.75B (2.75–2.75)	$3650	$15B (12–19)	$5 (4–7)
					$5669	$23B (18–29)	$8 (7–11)
	Targeted	1.40M (1.03–1.79)	0.003 (0.002–0.004)	0.45B (0.45–0.45)	$3650	$6B (4–7)	$12 (9–15)
					$5669	$8B (6–10)	$19 (14–23)

^a^ M = millions; ^b^ B = billions.

## Data Availability

The datasets used and/or analysed during the current study are available from the corresponding author on reasonable request.

## Supplementary Materials

The following are available online at https://www.mdpi.com/2076-393X/9/3/245/s1, Figure S1: Prevalence rate of tuberculosis in China in 2000, 2010 and 2050. Figure S2: Prevalence rate of tuberculosis in India in 2000, 2010 and 2050. Figure S3: Maximum total vaccine programme cost in the Policy baseline scenario. Figure S4: Maximum total cost per vaccine course in the Policy baseline scenario. Figure S5: Vaccination efficiency by target age group in the Policy scenario. Figure S6: Maximum total vaccine programme cost. Figure S7: Maximum total cost per vaccine course. Figure S8: Maximum total vaccine programme cost in the Policy scenario. Figure S9: Maximum total cost per vaccine course in the Policy scenario. Table S1: Mass vaccine campaigns (undiscounted). Table S2: Mass vaccine campaigns in the Policy scenario. Table S3: Mass vaccine campaigns in the Policy scenario (undiscounted).
