# Supplementary material for: Affordability of Adult Tuberculosis Vaccination in India and China: A Dynamic Transmission Model-Based Analysis
Source: Vaccines (Basel). 2021 Mar 11;9(3):245. doi: 10.3390/vaccines9030245 (PMC7998179; doi:10.3390/vaccines9030245)
Supplement: Supplementary file 1 [file vaccines-09-00245-s001.pdf]

Affordability of New Tuberculosis Vaccines in India and China: a Dynamic Transmission Model-based Analysis
Online Supplementary Materials

1 Additional results

1.1 Undiscounted Analysis

Total maximum undiscounted costs for the vaccine programme and per vaccine course are presented in Figure S1, Figure S2 and Table S1.

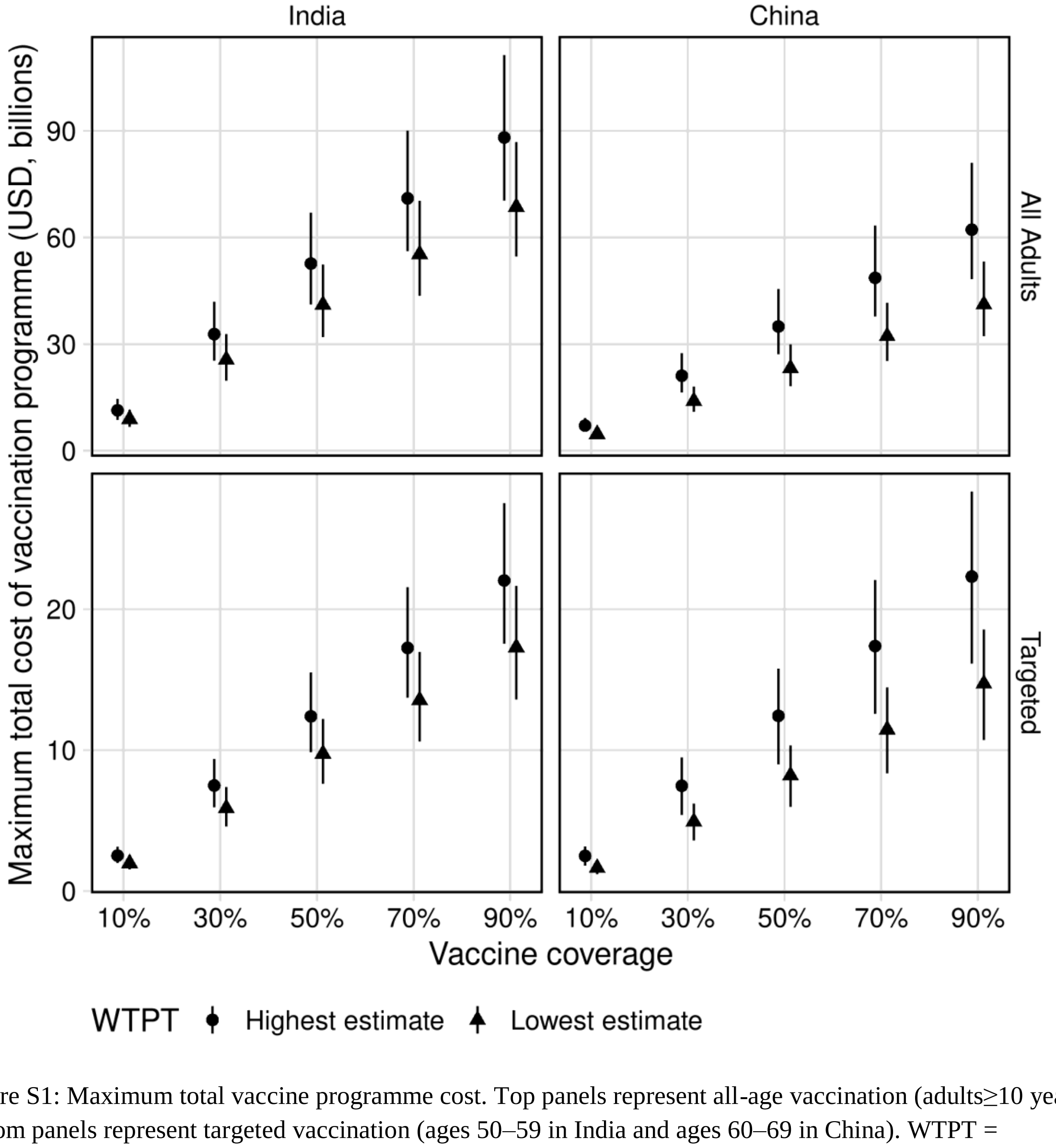

Figure S1: Maximum total vaccine programme cost. Top panels represent all-age vaccination (adults≥10 years); bottom panels represent targeted vaccination (ages 50–59 in India and ages 60–69 in China). WTPT = willingness to pay thresholds per Ochalek et al., estimated at $264 and $363 per DALY averted in India (lowest and highest estimates, respectively) and $3650 and $5669 per DALY averted in China (lowest and highest estimates, respectively). Costs undiscounted.

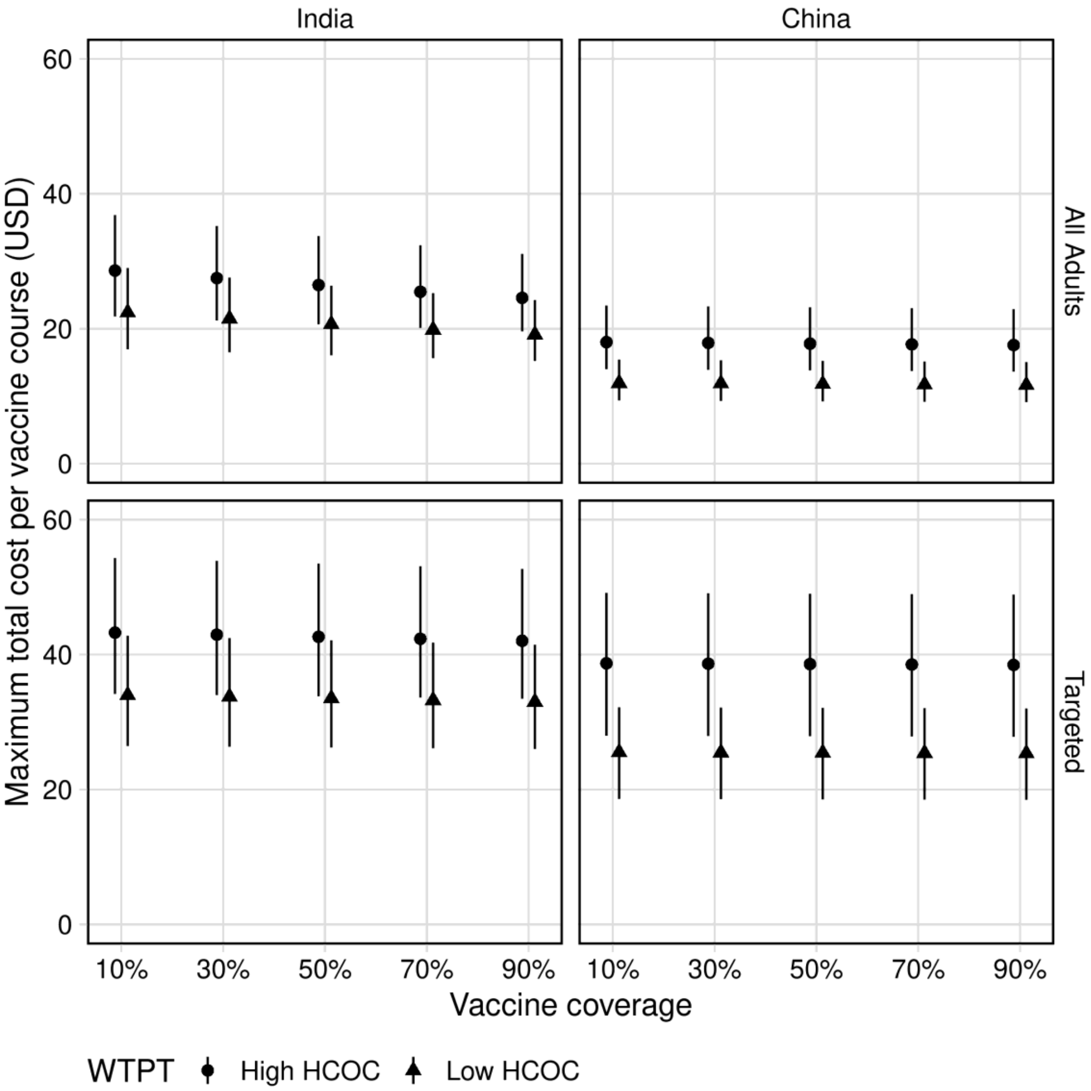

Figure S2: Maximum total cost per vaccine course. Top panels represent all-age vaccination (adults≥10 years); bottom panels represent targeted vaccination (ages 50–59 in India and ages 60–69 in China). WTPT = country-specific willingness to pay thresholds per Ochalek et al., estimated at $264 and $363 per DALY averted in India (lowest and highest estimates, respectively) and $3650 and $5669 per DALY averted in China (lowest and highest estimates, respectively). Costs undiscounted.

Table S1: Mass vaccine campaigns. Targeted vaccination in India was delivered to ages 50–59 and in China to ages 60–69. Results are aggregated over three campaigns delivered in 2027, 2037 and 2047. Averted DALYs and estimated net vaccine implementation costs are undiscounted. WTP: willingness to pay

Country	Campaign	Averted DALYs (total)[a]	Averted DALYs (per vaccine)	Vaccinations Delivered[b]	WTP	Maximum Total Cost of Vaccination Programme[b]	Maximum Total Cost per Vaccine Course
India	All Ages	158.21M (125.43–199.14)	0.057 (0.045–0.072)	2.79B (2.78–2.80)	264	$55B (44–70)	$20 (16–25)
					363	$71B (56–90)	$25 (20–32)
	Targeted	37.44M (29.15–46.41)	0.092 (0.071–0.114)	0.41B (0.41–0.41)	264	$14B (11–17)	$33 (26–42)
					363	$17B (14–22)	$42 (34–53)
China	All Ages	8.14M (6.21–10.76)	0.003 (0.002–0.004)	2.75B (2.75–2.75)	3,650	$32B (25–42)	$12 (9–15)
					5,669	$49B (38–63)	$18 (14–23)
	Targeted	2.93M (2.09–3.77)	0.007 (0.005–0.008)	0.45B (0.45–0.45)	3,650	$11B (8–14)	$25 (18–32)
					5,669	$17B (13–22)	$39 (28–49)

[a]M=millions

[b]B=billions

1.2 Age-specific prevalence

Model estimates of age-specific prevalence rates for tuberculosis in 2000, 2010 and 2050 are provided in Figures S3 (China) and S4 (India). In both countries, prevalence rate fell continuously from 2000 to 2050. In China (Figure S3), model estimates are shown against age-specific empirical estimates of TB prevalence rate derived from data underlying nationally representative TB prevalence surveys in 5-year age-groups in 2000 and 2010. We found good concordance between available data and model estimates in most age-groups, with slightly higher divergence in the elderly.

Figure S4 shows the age-specific prevalence rates estimated for India. There are no currently available nationally representative age-stratified estimates for TB prevalence, therefore corresponding empirical data are not shown. Similar to the pattern seen in China, the prevalence rate increased with age; however, the relative burden in the elderly (age≥65) was not as great in India.

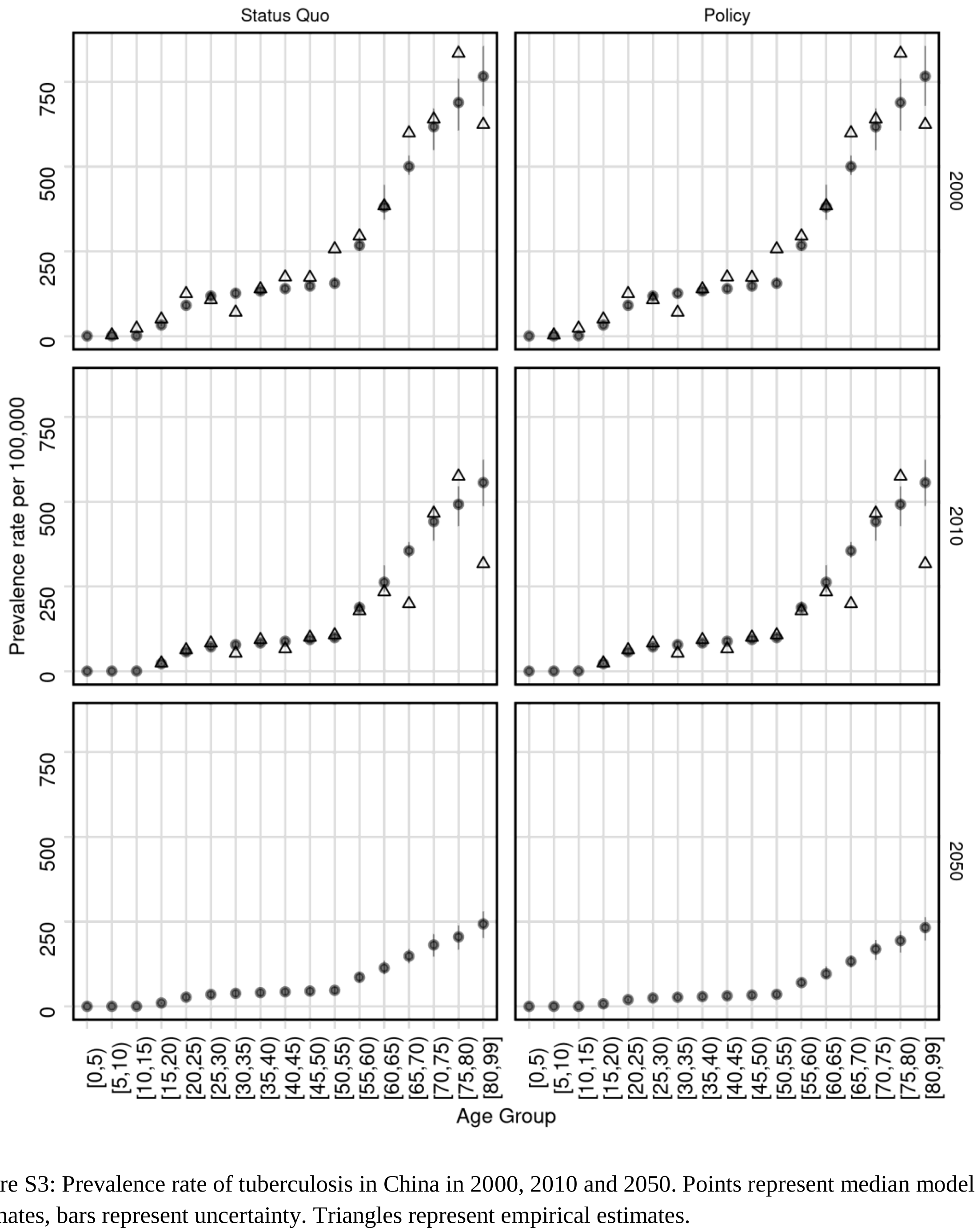

Figure S3: Prevalence rate of tuberculosis in China in 2000, 2010 and 2050. Points represent median model estimates, bars represent uncertainty. Triangles represent empirical estimates.

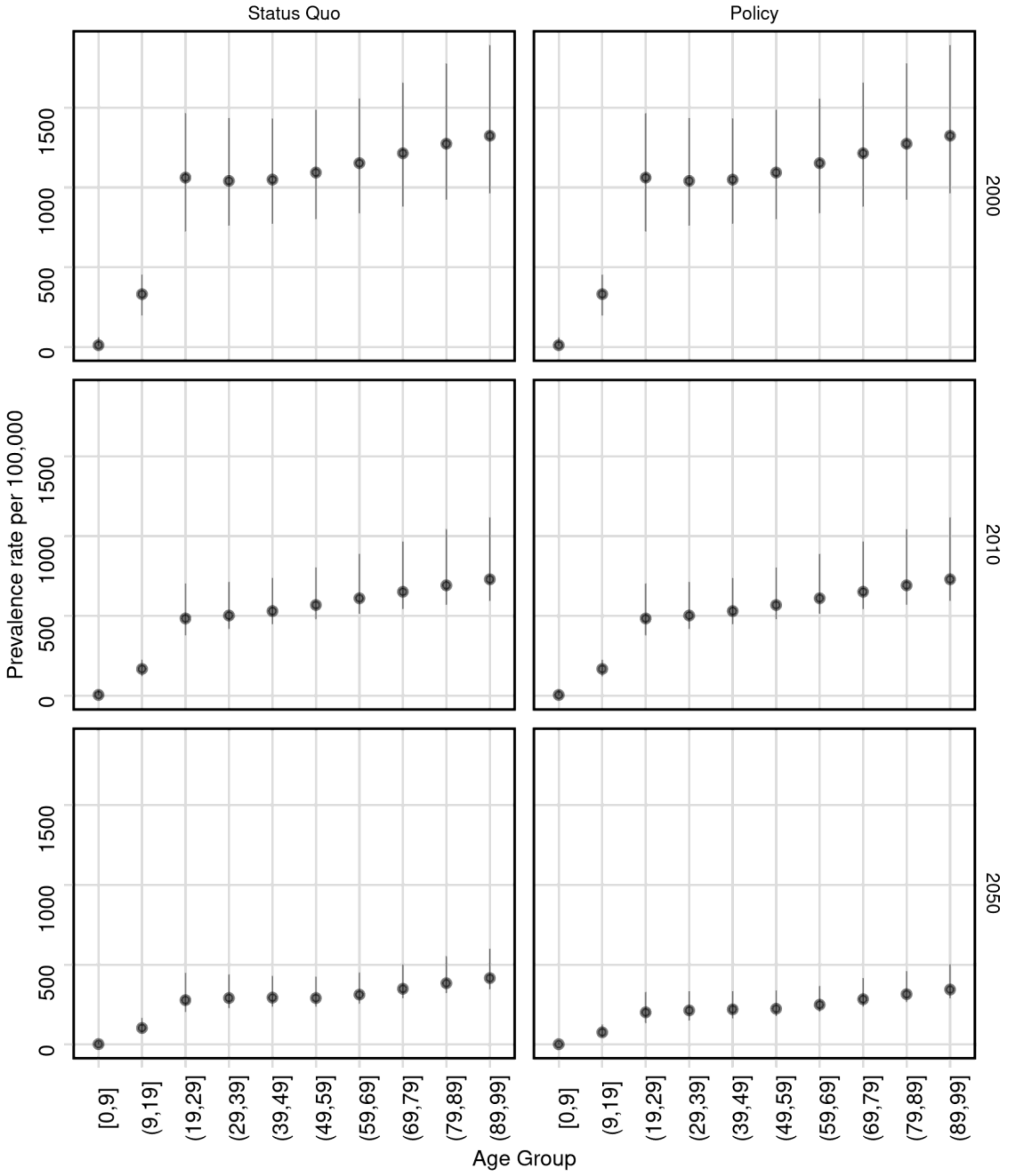

Figure S4: Prevalence rate of tuberculosis in India in 2000, 2010 and 2050. Points represent median model estimates, bars represent uncertainty.

1.3 Policy scenario

The estimated maximum total costs of the vaccination programme and estimated maximum total costs per vaccine course in the "Policy" scenario are presented in Figure S5, Figure S6 and Table S2. For both all age and targeted vaccination strategies, across all levels of coverage, estimated maximum total vaccine programme costs and maximum total costs per vaccine course were lower than in the converse baseline scenario without scale up of TB programme. Correspondingly, estimated averted DALYs per vaccine given (Figure S7) were found to be slightly lower. Total maximum undiscounted costs for the vaccine programme and per vaccine course in the Policy scenario are presented in Figure S8, Figure S9 and Table S3.

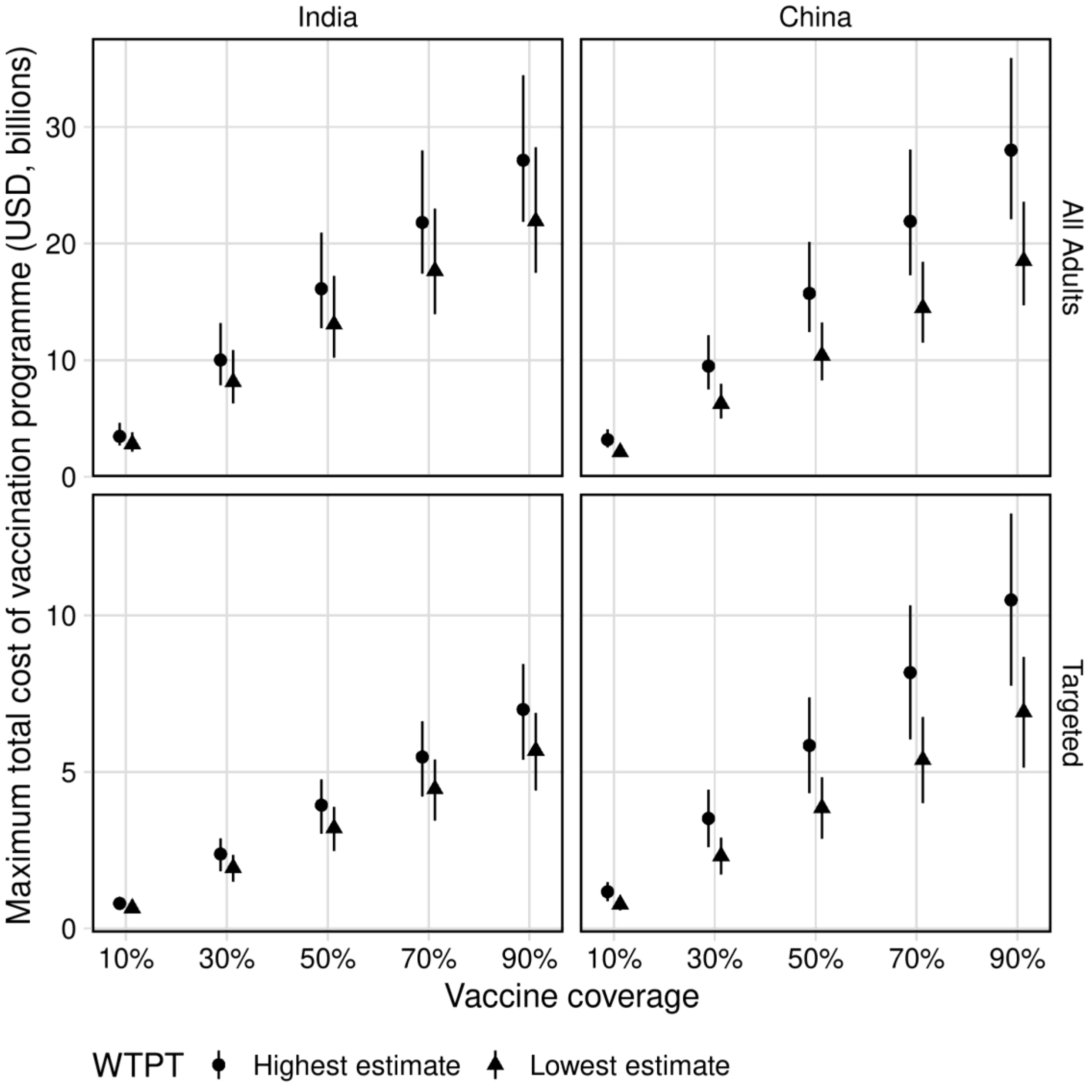

Figure S5: Maximum total vaccine programme cost in the Policy scenario. Top panels represent all-age vaccination (adults≥10 years); bottom panels represent targeted vaccination (ages 50–59 in India and ages 60–69 in China). WTPT = willingness to pay thresholds per Ochalek et al., estimated at \$264 and \$363 per DALY averted in India (lowest and highest estimates, respectively) and \$3650 and \$5669 per DALY averted in China (lowest and highest estimates, respectively). Costs discounted to 2018 values.

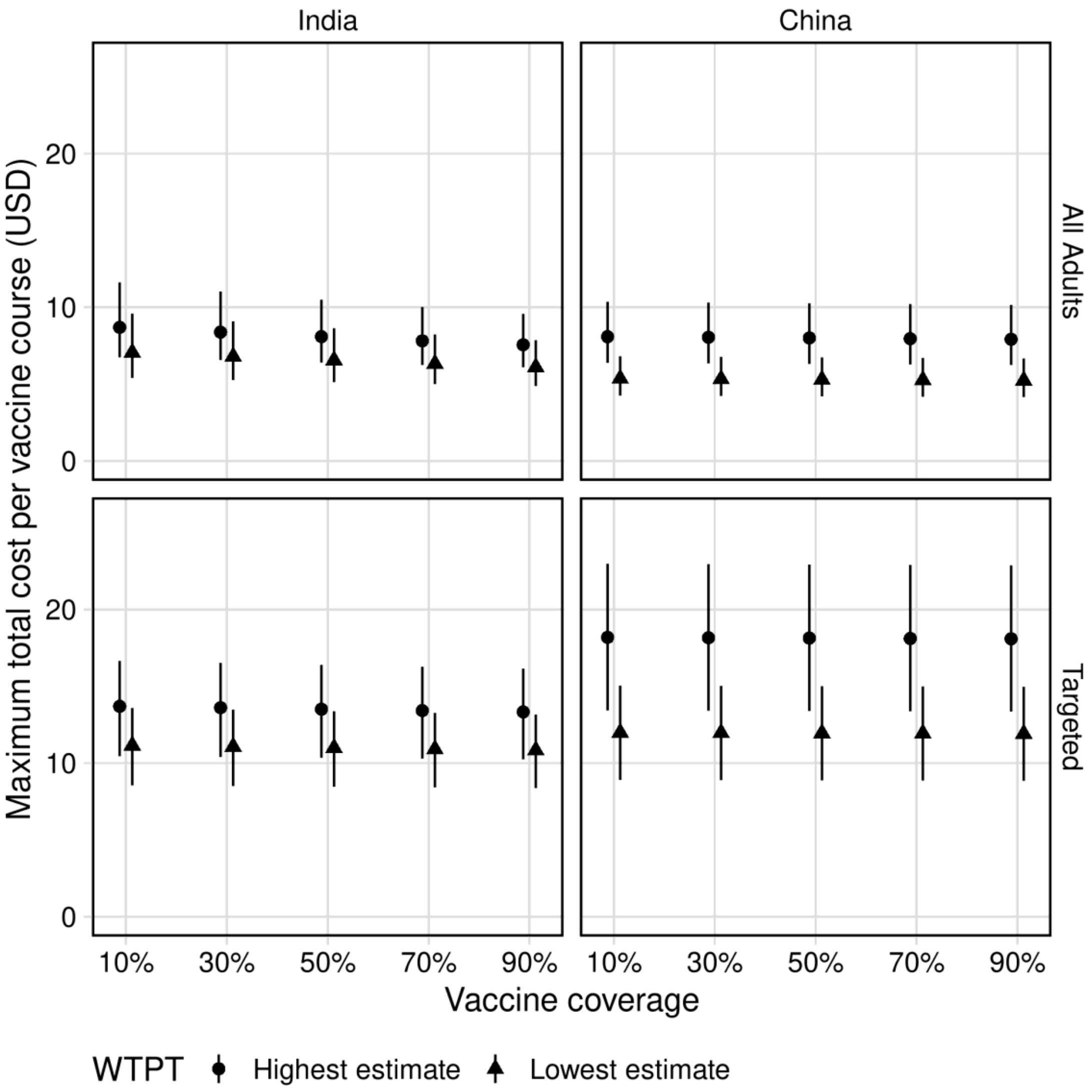

Figure S6: Maximum total cost per vaccine course in the Policy scenario. Top panels represent all-age vaccination (adults≥10 years); bottom panels represent targeted vaccination (ages 50–59 in India and ages 60–69 in China). WTPT = country-specific willingness to pay thresholds per Ochalek et al., estimated at $264 and $363 per DALY averted in India (lowest and highest estimates, respectively) and $3650 and $5669 per DALY averted in China (lowest and highest estimates, respectively). Costs discounted to 2018 values.

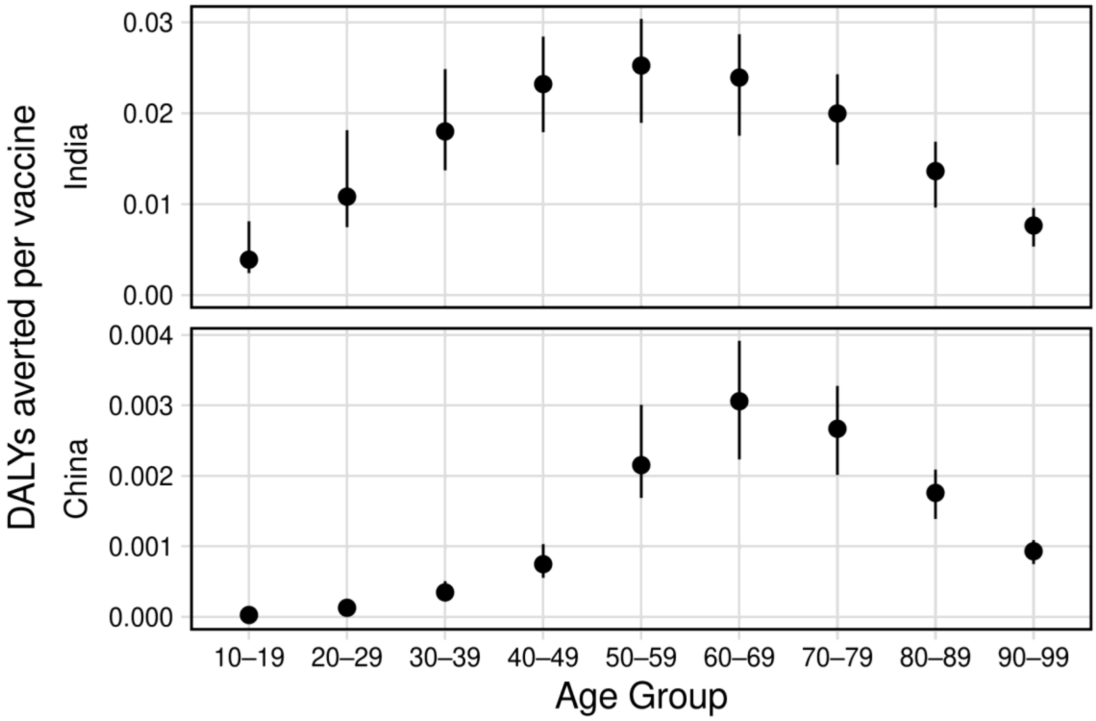

Figure S7: Vaccination efficiency by target age group in the Policy scenario. Efficiency is defined as the number of DALYs averted per vaccine delivered. Mass vaccine campaigns were deployed at 70% coverage to each age group.

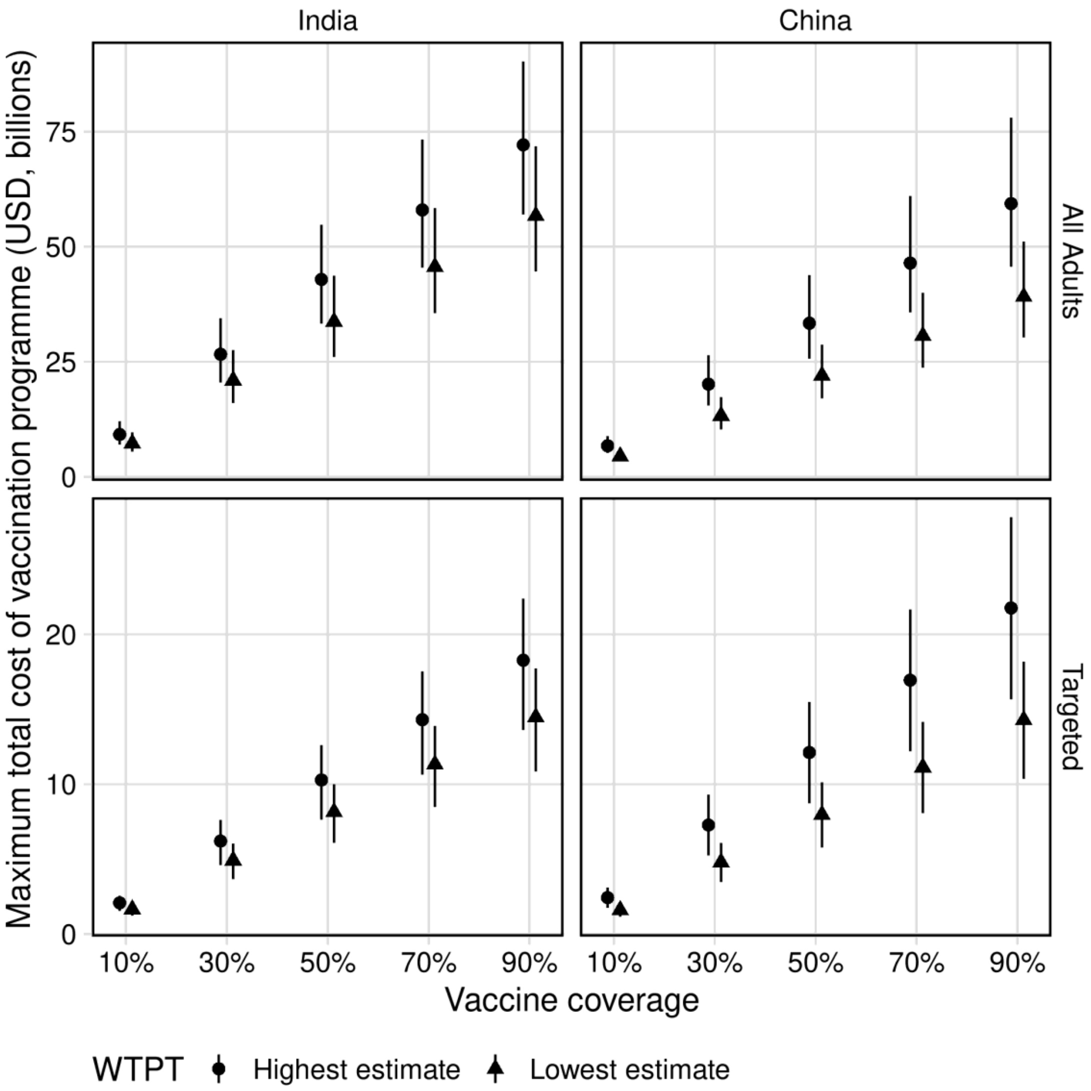

Figure S8: Maximum total vaccine programme cost in the Policy scenario. Top panels represent all-age vaccination (adults≥10 years); bottom panels represent targeted vaccination (ages 50–59 in India and ages 60–69 in China). WTPT = willingness to pay thresholds per Ochalek et al., estimated at \$264 and \$363 per DALY averted in India (lowest and highest estimates, respectively) and \$3650 and \$5669 per DALY averted in China (lowest and highest estimates, respectively). Costs undiscounted.

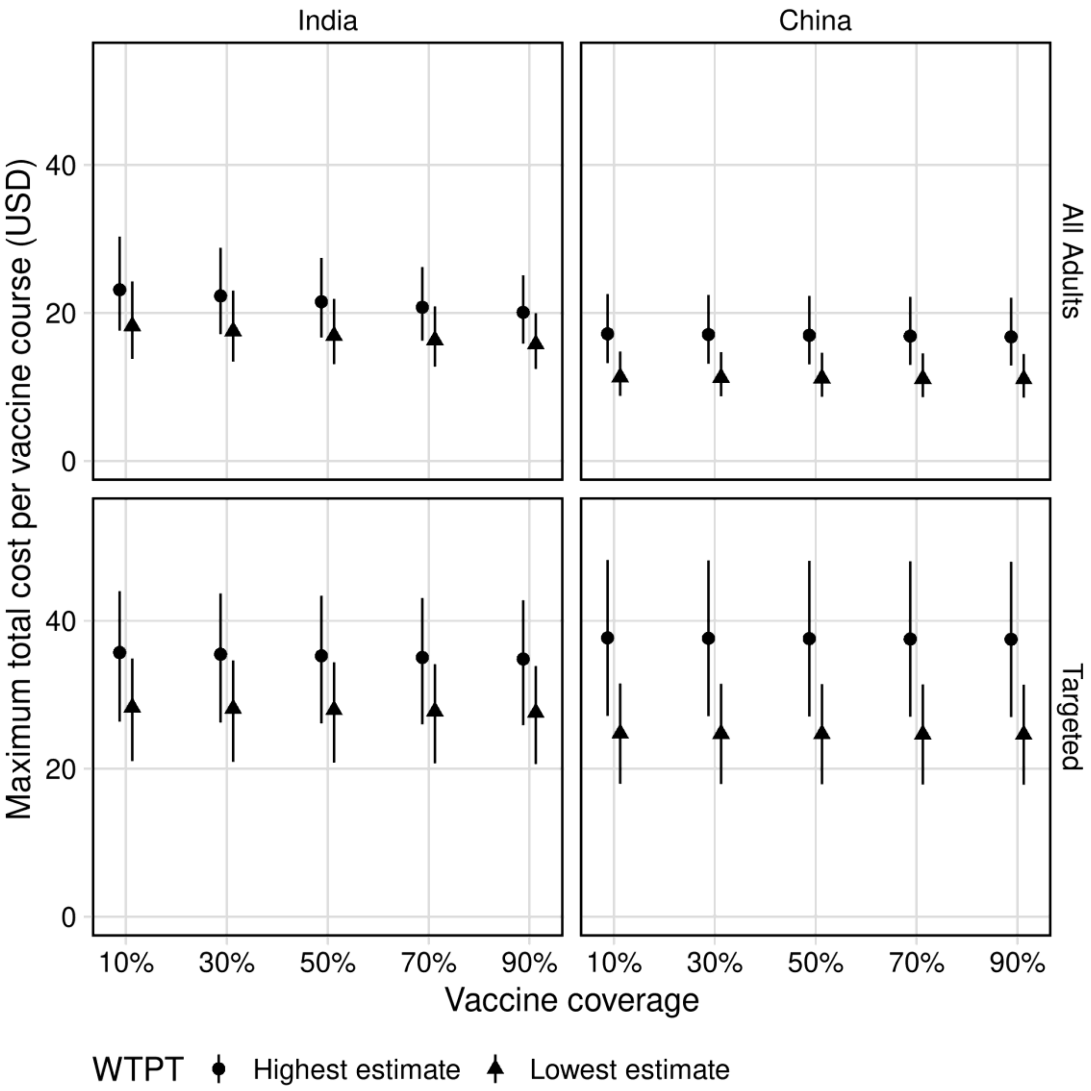

Figure S9: Maximum total cost per vaccine course in the Policy scenario. Top panels represent all-age vaccination (adults≥10 years); bottom panels represent targeted vaccination (ages 50–59 in India and ages 60–69 in China). WTPT = country-specific willingness to pay thresholds per Ochalek et al., estimated at $264 and $363 per DALY averted in India (lowest and highest estimates, respectively) and $3650 and $5669 per DALY averted in China (lowest and highest estimates, respectively). Costs undiscounted.

Table S2: Mass vaccine campaigns in the Policy scenario. Targeted vaccination in India was delivered to ages 50–59 and in China to ages 60–69. Results are aggregated over three campaigns delivered in 2027, 2037 and 2047. Averted DALYs and estimated net vaccine implementation costs are discounted to 2018 values at 3% per year. WTP: willingness to pay

Country	Campaign	Averted DALYs (total)[a]	Averted DALYs (per vaccine)	Vaccinations Delivered[b]	WTP	Maximum Total Cost of Vaccination Programme[b]	Maximum Total Cost per Vaccine Course
India	All Ages	42.23M (33.24–52.36)	0.015 (0.012–0.019)	2.79B (2.78–2.80)	264	$18B (14–23)	$6 (5–8)
					363	$22B (17–28)	$8 (6–10)
	Targeted	10.31M (7.75–12.36)	0.025 (0.019–0.030)	0.41B (0.41–0.41)	264	$4B (3–5)	$11 (8–13)
					363	$5B (4–7)	$13 (10–16)
China	All Ages	3.67M (2.86–4.77)	0.001 (0.001–0.002)	2.75B (2.75–2.75)	3,650	$14B (11–18)	$5 (4–7)
					5,669	$22B (17–28)	$8 (6–10)
	Targeted	1.38M (1.01–1.77)	0.003 (0.002–0.004)	0.45B (0.45–0.45)	3,650	$5B (4–7)	$12 (9–15)
					5,669	$8B (6–10)	$18 (13–23)

[a]M=millions

[b]B=billions

Table S3: Mass vaccine campaigns in the Policy scenario. Targeted vaccination in India was delivered to ages 50–59 and in China to ages 60–69. Results are aggregated over three campaigns delivered in 2027, 2037 and 2047. Averted DALYs and estimated net vaccine implementation costs are undiscounted. WTP: willingness to pay

Country	Campaign	Averted DALYs (total)[a]	Averted DALYs (per vaccine)	Vaccinations Delivered[b]	WTP	Maximum Total Cost of Vaccination Programme[b]	Maximum Total Cost per Vaccine Course
India	All Ages	125.21M (95.84–155.63)	0.045 (0.034–0.056)	2.79B (2.78–2.80)	264	$46B (36–58)	$16 (13–21)
					363	$58B (45–73)	$21 (16–26)
	Targeted	30.03M (21.80–37.18)	0.074 (0.053–0.091)	0.41B (0.41–0.41)	264	$11B (8–14)	$28 (21–34)
					363	$14B (11–18)	$35 (26–43)
China	All Ages	7.82M (5.95–10.42)	0.003 (0.002–0.004)	2.75B (2.75–2.75)	3,650	$31B (24–40)	$11 (9–15)
					5,669	$46B (36–61)	$17 (13–22)
	Targeted	2.87M (2.05–3.72)	0.006 (0.005–0.008)	0.45B (0.45–0.45)	3,650	$11B (8–14)	$25 (18–31)
					5,669	$17B (12–22)	$38 (27–48)

[a]M=millions

[b]B=billions
